# Supplementary material for: Information needs among women taking part in primary HPV screening in England: a content analysis
Source: BMJ Open. 2020 Dec 15;10(12):e044630. doi: 10.1136/bmjopen-2020-044630 (PMC7745520; doi:10.1136/bmjopen-2020-044630)
Supplement: Supplementary data [file bmjopen-2020-044630supp002.pdf]

Supplementary File 1 – additional information on HPV primary screening in England

Figure 1 – flow chart of HPV primary screening in the English HPV primary screening pilot sites

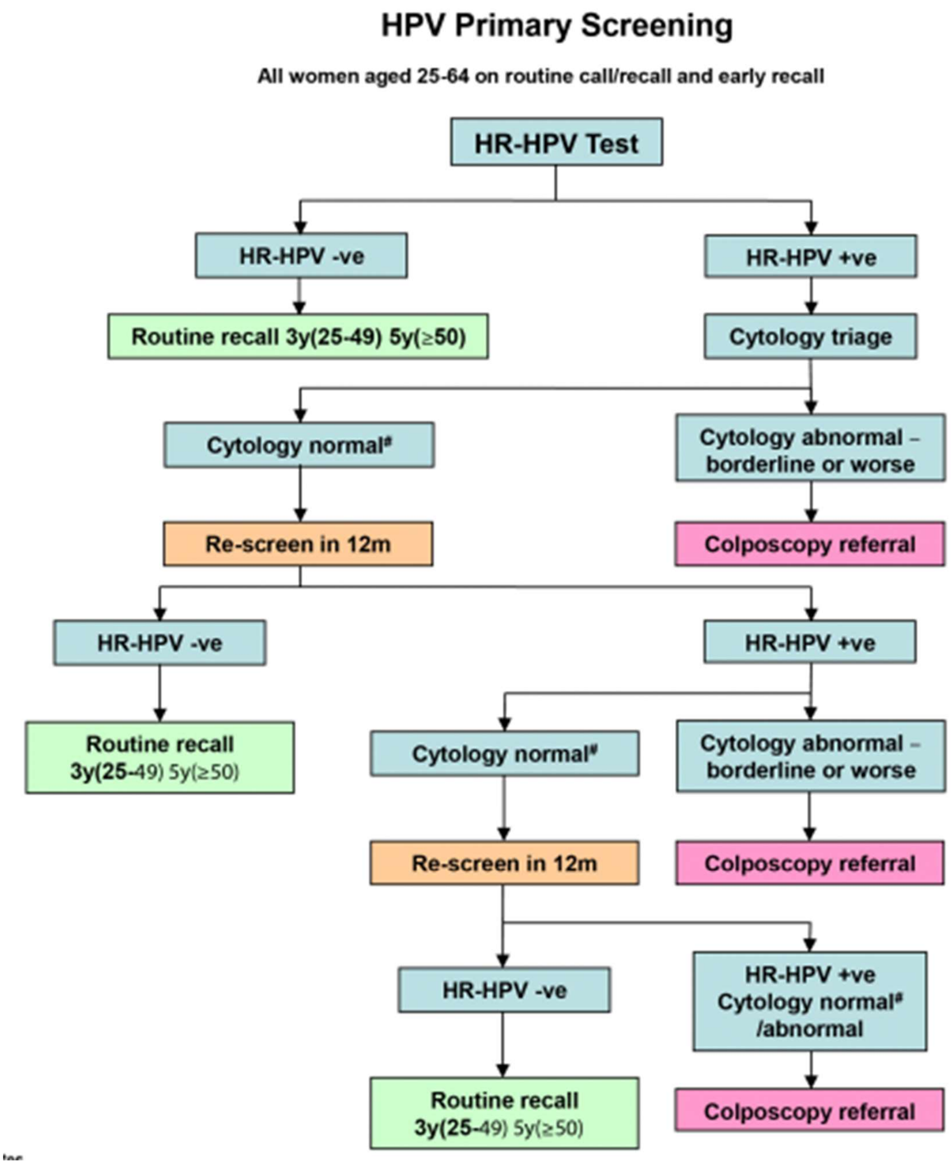

Figure from https://phescreening.blog.gov.uk/2017/06/14/what-gps-need-to-know-about-the-introduction-of-primary-hpv-testing-in-cervical-screening/

Additional information

Cervical screening in England is organised as a national call-recall programme and is free at the point of use. From the age of 24.5 years, women are invited for screening by letter every three years until age 49 years and every five years from age 49-64 years. The invitation is sent in the post and includes a leaflet with further information on cervical cancer, HPV and the screening procedure (https://assets.publishing.service.gov.uk/government/uploads/system/uploads/attachment_data/file/871893/Cervical_screening_helping_you_decide.pdf).

Screening is usually carried out by a nurse at a woman's general practice (GP) surgery but may sometimes be delivered at a sexual health or well-woman clinic. Women receive their screening results by post and if they are

referred for colposcopy, they also receive a colposcopy leaflet (https://www.gov.uk/government/publications/cervical-screening-colposcopy/cervical-screening-having-a-colposcopy).

As HPV primary screening was first piloted in certain areas and then rolled out gradually across England over the course of a year, there was no public health campaign to launch HPV primary screening.

The current study took place in the context of the HPV primary screening pilot, where women were sent the following information about HPV testing with their invitation.

NHS Cervical Screening Programme

HPV primary screening

This leaflet tells you about HPV primary screening. This is a test carried out on the sample of cells we take during cervical screening.

- HPV stands for 'human papillomavirus'.
- Primary screening means that it is the first test carried out on the sample of cells from your cervical screening.

HPV primary screening makes no difference to how your screening sample is taken.

For more information about going for screening, please read our leaflet *NHS cervical screening*.

Why have I been sent this leaflet?

You live in an area where HPV primary screening is being used in the NHS Cervical Screening Programme. This isn't happening everywhere in England yet.

What is HPV?

The human papillomavirus (HPV) is very common. Most women get it at some point in their lives. There are many types of HPV, and most of them clear up by themselves without causing any problems. However, some types can cause cells in the cervix to change and become abnormal. These abnormal changes can, if left untreated, go on to turn into cervical cancer.

HPV is easily passed on during intimate sexual contact between partners; between men and women and between partners of the same sex. The virus has no symptoms. This means that you or a partner may have had HPV for many months or years without knowing it.

March 2013

What is HPV primary screening?

Once your cervical screening sample is sent to the laboratory, the first test carried out on it will be to look for HPV. If HPV is found, your sample will also be looked at for abnormal cells. Looking for abnormal cells is called 'cytology'. Cytology is still the test used for most cervical screening at the moment. If you don't have HPV, then it is extremely unlikely that you will have any abnormal cervical cells.

HPV primary screening should benefit women because:

- more abnormal cervical cells will be picked up
- women without HPV can be reassured that they are at extremely low risk of developing cervical cancer.

Are all women being offered HPV primary screening?

Not yet. We are starting HPV primary screening in a few areas from 2013. This is to help us plan how this change to the screening programme can be put in place across the whole of England.

In places where HPV primary screening isn't being used yet, women will either

- not have an HPV test or
- have an HPV test only after their sample has been checked for abnormal cells (cytology).

What screening results might I get?

There are three main types of results from HPV primary screening.

No HPV found (HPV negative)

If no HPV is found, then no further tests will be carried out. If you don't have HPV, then it is highly unlikely that you will have any abnormal cervical cells. Even if you did, it would be extremely unlikely that they would cause a problem. You will simply be called back for screening again in three or five years' time (depending on your age).

HPV found (HPV positive) but no abnormal cervical cells found

If HPV is found, the sample will also be tested for abnormal cervical cells. If none are found, your result will say you have HPV, but no abnormal cells. You will be asked to come back for screening again in 12 months' time. This is so we can check that the HPV has been cleared by your immune system. If it hasn't cleared, you may be at greater risk of developing abnormal cervical cells.

HPV found (HPV positive) and abnormal cervical cells found

There are several grades of abnormal cells. Some are more serious than others. You can read about this in our leaflet *What your abnormal result means*. If you have HPV *and* any grade of abnormal cervical cells, then you will be referred for colposcopy. Colposcopy is a closer examination of the cervix. It is carried out in much the same way as cervical screening. For more information, you can read our leaflet *The colposcopy examination*.

It is also possible to have an 'inadequate' result. This is when the laboratory cannot get an HPV test result from your sample, or cannot see if abnormal cells are present or not. If you have an inadequate result, you may be asked to have cervical screening again in three months' time. The delay is so that there are enough cells again to get a sample from.

Can I get treatment for HPV?

No, there isn't a treatment to get rid of the virus. For most women, their immune system will get rid of HPV – like getting rid of a common cold. But we can treat abnormal cervical cells, especially if they are found early on. Most types of cervical cancer take a long time to develop. Treating abnormal cells early on means that cervical cancer can be prevented.

Where can I get more information?

If you would like more information about HPV primary screening, or anything else in this leaflet, you can

- talk to the nurse at your GP practice;
- visit our website at www.cancerscreening.nhs.uk/cervical

Copies of the leaflets mentioned can also be downloaded from our website.

A large print version of this leaflet is available at www.cancerscreening.nhs.uk/cervical
