# Supplementary material for: Information needs among women taking part in primary HPV screening in England: a content analysis
Source: BMJ Open. 2020 Dec 15;10(12):e044630. doi: 10.1136/bmjopen-2020-044630 (PMC7745520; doi:10.1136/bmjopen-2020-044630)
Supplement: Supplementary data [file bmjopen-2020-044630supp001.pdf]

Supplementary Figure 2: Overall response rate and proportion of women leaving a free-test response during the course of the study

Sent questionnaire	Returned questionnaire[a]	Free-text response
HPV-negative n=1229	n=248 (20.2%)	n=65 (26.2%)
HPV-positive, cytology normal n=1198	n=258 (21.5%)	n=129 (50.0%)
HPV-positive, cytology abnormal n=810	n=170 (21.0%)	n=67 (39.4%)
HPV-persistent (at 12 months) n=663	n=179 (27.0%)	n=91 (50.8%)
HPV-cleared (at 12 months) n=262	n=66 (25.2%)	n=29 (43.9%)
Control group (not tested for HPV) n=1332	n=206 (15.5%)	EXCLUDED

[a] Excluding questionnaires that were returned late or where participants were an ineligible age (n=21).
